# MSC-derived exosomes for hemorrhagic stroke: preclinical evidence and translational challenges

**DOI:** 10.3389/fneur.2026.1711050

**Published:** 2026-04-29

**Authors:** Ivonne Salinas, Laura Vela, Shabnam Santos, Ariel Moncayo, Kevin Moreno, Auki Guaillas, Ramiro F. Diaz, Andrés Caicedo

**Affiliations:** 1 Universidad San Francisco de Quito USFQ, Colegio de Ciencias de la Salud, Escuela de Medicina, Quito, Ecuador; 2 Universidad San Francisco de Quito USFQ, Escuela de Medicina Veterinaria, Quito, Ecuador; 3 Mito-Act Research Consortium, Quito, Ecuador; 4 Universidad San Francisco de Quito USFQ, Instituto de Investigaciones en Biomedicina iBiomed, Quito, Ecuador; 5 Universidad San Francisco de Quito USFQ, USFQ Space Front, Quito, Ecuador; 6 Homeos Health Research, Quito, Ecuador

**Keywords:** exosomes, extracellular vesicles, hemorrhagic stroke, mesenchymal stem/stromal cells, neuroinflammation, neuroregeneration, angiogenesis, translational medicine

## Abstract

Hemorrhagic stroke, caused by bleeding into the brain parenchyma or subarachnoid space, accounts for 10–20% of cerebrovascular events worldwide. It is classified as intracerebral hemorrhage (ICH) or subarachnoid hemorrhage (SAH). Despite distinct etiologies, both forms initiate a shared injury cascade marked by metabolic failure, mitochondrial dysfunction, oxidative stress, cytotoxic edema, and progressive neuronal loss. Current guidelines prioritize time-sensitive, neuroprotective measures aimed at acute stabilization and complication prevention. However, these interventions remain largely supportive and fail to directly address the sustained secondary injury processes that underlie long-term neurological disability. In this Perspective, we focus on mesenchymal stem/stromal cell (MSCs)–derived exosomes as a promising cell-free therapeutic strategy with distinct advantages over MSC-based therapies. We first provide an overview of the key mechanisms of neuronal injury in hemorrhagic stroke, distinguishing early brain injury from delayed, secondary damage. We then define exosomes within the broader extracellular vesicle landscape and explain why MSC-derived exosomes are emphasized as principal mediators of MSC paracrine effects. Finally, we synthesize preclinical evidence showing that exosomes can attenuate neuroinflammation, limit apoptosis, and promote angiogenesis and neurogenesis, with associated improvements in functional recovery in experimental stroke models. We also highlight unresolved challenges identified in the current literature, including uncertainties surrounding therapeutic timing, dosing strategies, vesicle heterogeneity, and the need for improved *in vivo* tracking and mechanistic resolution. As the field advances, addressing these critical issues will be essential for translating MSC-derived exosomes into effective therapies for hemorrhagic stroke.

## Introduction

Hemorrhagic stroke, a devastating cerebrovascular event caused by blood extravasation into the brain parenchyma or subarachnoid space, accounts for ≈10–20% of all cerebrovascular events worldwide, with a global incidence of 12–15 cases per 100,000 inhabitants annually (1). Clinically, it is categorized into intracerebral hemorrhage (ICH) and subarachnoid hemorrhage (SAH) (2). ICH most often results from chronic hypertension that weakens penetrating cerebral arteries, although ruptured aneurysms, arteriovenous malformations, and vasculitis also contribute (3). SAH typically arises from head trauma or rupture of saccular aneurysms in the circle of Willis (4). Despite these distinct etiologies, both forms converge on a common pathophysiological cascade: disruption of neuronal metabolism, mitochondrial dysfunction, cytotoxic edema, oxidative stress, and ultimately neuronal death (5). Although the overall incidence of stroke has declined in the last three decades, recent data indicate that hemorrhagic stroke constitutes an increasing proportion of all strokes, and its mortality rates have risen compared with ischemic stroke (6). These trends highlight its disproportionate clinical burden and underscore the urgent need for strategies to reduce morbidity and mortality.

Morbidity in stroke is primarily consequent of neural cell loss, which is why current guidelines provided by the American Stroke Association (ASA) address treatment in a time sensitive and neuroprotective fashion (7). However, current management strategies for hemorrhagic stroke fail to address neural cell decay that occurs both immediately and during the prolonged post-stroke period (7). Understanding the mechanisms of cellular brain injury in hemorrhagic stroke elicits an opportunity to explore novel therapeutic techniques that could significantly reduce sequels and overall morbidity in the post-stroke period (8).

Hemorrhagic stroke inundates the brain parenchyma with blood, hemoglobin, and iron-rich degradation products, initiating a robust inflammatory cascade that amplifies neuronal death. Given the complexity and severity of these processes, no single pharmacological agent currently offers sufficient control over the multifactorial damage. In the search for novel therapeutic strategies, mesenchymal stem/stromal cells (MSCs) have shown promise in mitigating injury through their paracrine effects—attenuating neuroinflammation, inhibiting apoptosis, promoting angiogenesis and neurogenesis (9–11). Importantly, emerging evidence indicates that exosomes, released by virtually all cell types and particularly enriched in MSCs, are principal mediators of these reparative benefits. Exosomes encapsulate bioactive lipids, proteins, and regulatory RNAs that reflect the molecular profile of their parent cells, and could repair or cross the blood–brain barrier (BBB) to reach injured tissue at varying rates and through vesicular-mediated mechanisms (12–16). Compared with whole-cell MSC therapies, exosomes exhibit superior colloidal stability, markedly reduced immunogenicity, teratome formation and eliminate risks such as microvascular occlusion. These advantages position exosomes as a safer and potentially more effective intervention for hemorrhagic stroke (17–19).

In this Perspective, we explore exosomes as cell-free carriers of regenerative signals with the potential to improve recovery after hemorrhagic stroke. By modulating neuroinflammation, apoptosis, angiogenesis, and neuroplasticity, exosomes—particularly those derived from MSCs—represent a versatile, potentially off-the-shelf therapeutic platform that targets key biological processes not addressed by current standard care. Building on a growing body of preclinical evidence demonstrating neurorestorative effects in the subacute and chronic phases after ICH, we first situate exosomes within the broader extracellular vesicle landscape and discuss their relevance as therapeutic candidates. We then outline the cellular and molecular mechanisms driving injury in hemorrhagic stroke to frame opportunities for translation. Finally, we synthesize mechanistic evidence supporting exosome-mediated repair and highlight critical challenges that must be addressed to enable clinical translation, including manufacturing and characterization standards, dosing and delivery strategies, biodistribution and BBB penetration, safety considerations, and regulatory pathways.

## Understanding neuronal injury in hemorrhagic stroke

**Figure 1:**
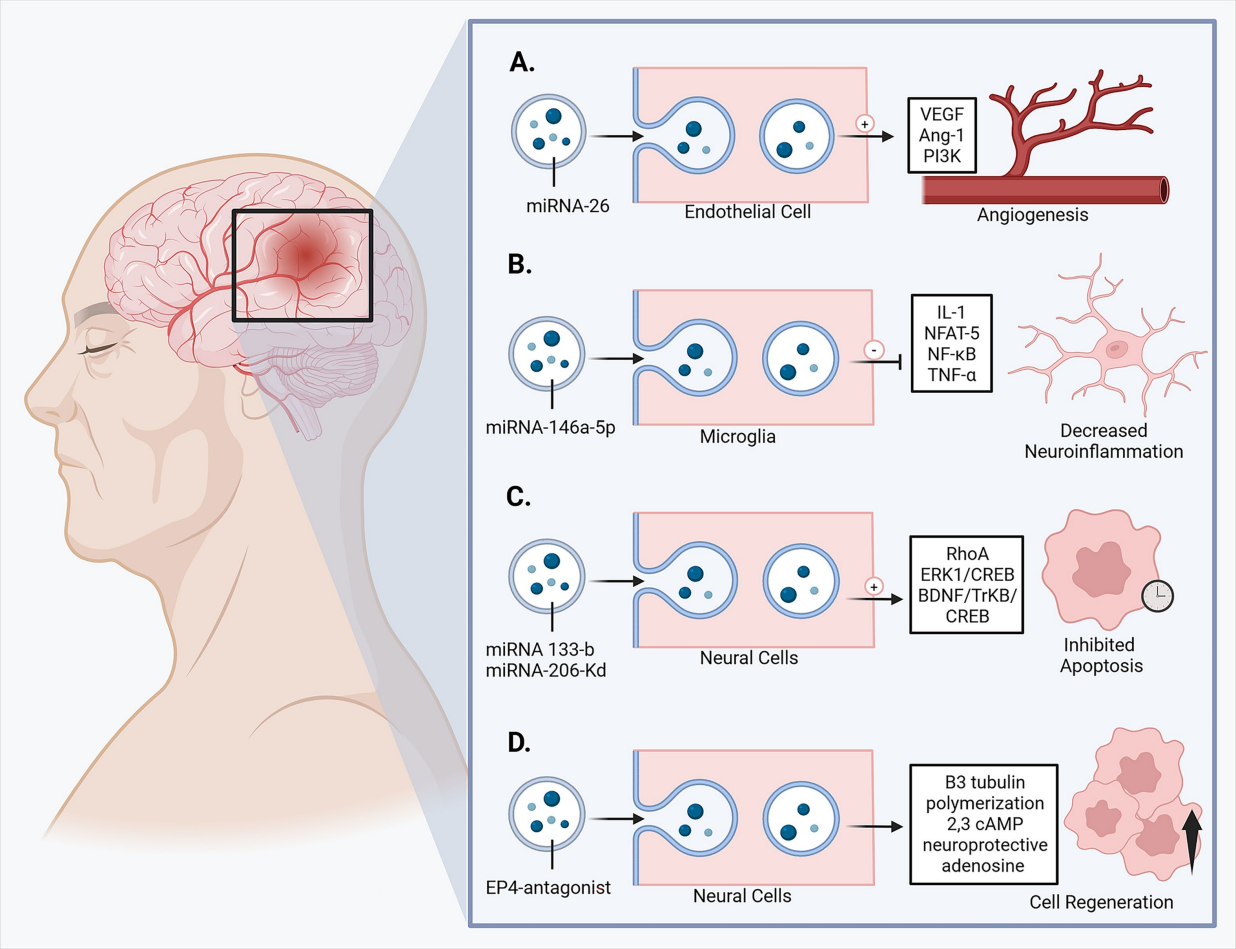
Exosome therapeutic mechanisms of action in hemorrhagic stroke. **(A)** Angiogenesis, **(B)** decreased neuroinflammation, **(C)** inhibited apoptosis, **(D)** cell regeneration. Created in https://BioRender.com

Hemorrhagic stroke initiates a complex cascade of cellular and molecular events that result in widespread brain damage and contribute to its high morbidity and mortality. To evaluate the therapeutic potential of exosomes in this context, it is crucial to first understand the underlying pathophysiology of neuronal injury. Exosome efficacy may depend heavily on the timing of administration, as different stages of injury involve distinct, but interconnected, mechanisms that drive secondary brain damage and hinder recovery. These processes are typically classified into early and late phases, each presenting unique therapeutic windows and targets for intervention (Figure 1).

### Early brain injury

Primary injury in hemorrhagic stroke occurs immediately following the rupture of a blood vessel, as extravasated blood exerts direct mechanical pressure on brain tissue. This leads to acute parenchymal disruption, neuronal necrosis, and a rapid rise in intracranial pressure (20, 21). One of the earliest consequences of this pressure buildup is cerebral vasoconstriction, accompanied by endothelial activation. These changes promote thrombus formation and contribute to secondary ischemic injury. Simultaneously, the degradation of extravasated blood components—such as hemoglobin and iron—triggers catalytic reactions that generate reactive oxygen species (ROS) and initiate a neuroinflammatory response. These damaging processes persist and intensify during the later phases of injury (22).

### Late brain injury

Secondary injury evolves over hours to days following the initial hemorrhage, involving a network of interrelated pathophysiological processes. Progressive neuronal and glial damage is driven by hemoglobin and iron toxicity, thrombin signaling, excitotoxic neurotransmitter release, oxidative and nitrosative stress, inflammation and microglial activation, perihematomal edema, apoptosis/necrosis, and white-matter injury that together worsen functional outcomes (23).

### Oxidative stress

Hemorrhagic stroke induces excessive production of ROS from multiple sources, including mitochondrial dysfunction, erythrocyte lysis, hypoxic neurons, and activation of microglia and neutrophils. The accumulation of ROS, like superoxide radicals, hydrogen peroxide, and hydroxyl radicals, exacerbates oxidative damage, disrupts the BBB, and triggers lipid peroxidation, ultimately leading to neuronal apoptosis and necrosis (24, 25). Furthermore, due to the extremely short lifespan of free radicals, their direct detection is challenging. Consequently, the levels of antioxidants are typically measured as an indirect indicator. The body defends itself against ROS through both enzymatic mechanisms, such as those involving superoxide dismutase and glutathione peroxidase, and non-enzymatic mechanisms, which include antioxidants like vitamin E, copper, vitamin C, selenium, and others (25, 26).

### Neuroinflammation

The presence of blood and its degradation products in the brain parenchyma provokes a robust inflammatory response. Upon vascular rupture, damaged endothelial cells become activated, leading to the release of von Willebrand factor (vWF) from Weibel-Palade bodies. This process subsequently promotes platelet activation, facilitates leukocyte recruitment, and contributes to the phenomenon of capillary no-reflow (27).

Some glial cells such as microglia and astrocytes are the first cells to respond when there is a bleeding, and they do so by secreting pro-inflammatory cytokines (e.g., TNF-*α*, IL-1β, IL-6) and chemokines, perpetuating tissue damage and exacerbating cerebral edema (24). In addition, damaged neurons release danger-associated molecular patterns (DAMPs), like high mobility group box-1 (HMGB1) protein, which leads to the activation of the inflammasome and the nuclear factor κB (NF-κB) signaling pathway via interaction of Toll-like receptor (TLR) 2 and TLR4, exacerbating the production and release of pro-inflammatory cytokines (27).

Furthermore, macrophages and neutrophils infiltrate the affected area, where they engage in complex interactions that facilitate the presentation of antigens derived from necrotic tissue. This process subsequently activates T cells, contributing to the disruption of the BBB and exacerbating cerebral injury (20).

### Neural death pathways

Cell death begins as early as ten minutes after SAH, with the appearance of apoptotic bodies and neuronal necrosis (28) Apoptosis and necrosis differ fundamentally: apoptosis is an energy-dependent, programmed form of cell death, whereas necrosis is passive and energy-independent. When cells become energy-depleted after SAH, ionic pumps—particularly sodium and calcium pumps—fail. This ionic imbalance leads to cellular swelling, activation of proteases and phospholipases, and ultimately a transition from apoptosis to necrosis as ATP levels drop. Necroptosis represents an additional, regulated form of necrosis. Unlike apoptosis, which depends on active caspase-8, necroptosis is triggered when caspase-8 is inhibited. It is commonly activated by tumor necrosis factor-*α* (TNF-α) and is mediated through a well-defined signaling complex: Receptor-Interacting Protein Kinase 1 (RIPK1) and Receptor-Interacting Protein Kinase 3 (RIPK3) assemble to form the necrosome, which then phosphorylates and activates Mixed Lineage Kinase Domain-like protein (MLKL). Activated MLKL disrupts the plasma membrane, causing cell rupture and inflammation, a hallmark distinguishing necroptosis from apoptosis (29).

Another form of regulated necrosis is pyroptosis, which is activated in the context of strong inflammatory responses. During pyroptosis, the inflammasome triggers the activation of caspase-1, leading to plasma membrane permeabilization and subsequent necrotic cell death (29). Autophagy is another mechanism involved after SAH. Cellular hypoxia and nutrient deprivation stimulate this process by activating nuclear factor erythroid 2–related factor 2 (Nrf2) and B-cell lymphoma 2 (Bcl-2), ROS, and inhibiting mammalian target of rapamycin (mTOR) signaling. mTOR inhibition facilitates autophagosome formation through activation of phosphoinositide 3-kinase (PI3K) and adenosine monophosphate–activated protein kinase (AMPK). Through these pathways, autophagy helps limit inflammation and preserve cellular homeostasis in the injured brain (30). A particularly important form of neuronal death initiated after hemorrhagic stroke is ferroptosis. Blood-derived products—such as hemoglobin, heme, and iron—elicit strong inflammatory responses and exert direct toxic effects on neurons, astrocytes, and microglia. Heme promotes inflammatory signaling and drives neutrophil recruitment and activation. Iron, through glutamate release, ROS generation, and lipid peroxidation, induces profound oxidative stress that culminates in ferroptotic cell death (31).

Preventing secondary injury after hemorrhagic stroke remains a major challenge, particularly because neuronal and glial cells are highly vulnerable to hemoglobin- and iron-induced toxicity. These blood-derived products trigger intense oxidative stress and neuroinflammation, two key drivers of progressive brain damage. Multiple molecular pathways regulate these processes, and exosomes play an important modulatory role within them. Growing evidence suggests that the administration of exosomes holds promise for counteracting the inflammatory cascade associated with hemorrhagic stroke. By modulating immune responses, limiting microglial overactivation, and reducing oxidative stress, exosomes may help slow or prevent the progression of secondary brain injury. However, timing is critical. Early intervention could theoretically reduce acute damage, but strong experimental evidence supporting exosome delivery immediately after injury is still limited. As current treatments for hemorrhagic stroke, such as blood pressure control and the cautious use of antithrombotic therapy, continue to show significant limitations, the biological and restorative properties of exosomes may offer an innovative therapeutic opportunity. These potential mechanisms and therapeutic benefits will be described in detail in the following section of this review.

## Current management of hemorrhagic stroke

The treatment landscape for hemorrhagic stroke is multifaceted, involving both medical and surgical interventions. Early management focuses on acute stabilization and prevention of secondary injury, particularly during the first minutes to hours when outcomes are most modifiable. In the acute phase, the priority is to ensure airway, breathing, and circulation (ABC), followed by rapid implementation of neuroprotective measures to limit further neurological damage. Control of active bleeding, often by lowering blood pressure, and the prevention of complications such as elevated intracranial pressure (ICP), seizures, and cerebral vasospasm are central components of care (7).

Despite these measures, the current treatment options for SAH and ICH remain insufficient for improving long-term neurological outcomes (32). Standard therapies primarily aim to prevent further deterioration rather than restore lost function. This therapeutic gap has driven growing interest in innovative strategies, such as exosome-based interventions delivered during the early therapeutic window, to enhance recovery and promote neuronal repair (8).

### Hemostatic therapies

Hemostatic therapies are essential for preventing hematoma expansion. Their main goal is to avoid further complications related to the event itself once etiology has been established. This is critical because hematoma size strongly predicts mortality and functional outcome (33). Emerging adjunctive approaches include medical gas therapies, such as hyperbaric oxygen and hydrogen sulfide. These gases have demonstrated neuroprotective effects by modulating inflammation, oxidative stress, and apoptosis; however, potential neurotoxic effects warrant careful investigation (34). Their role remains exploratory but promising.

### Blood pressure management

Hypertension is one of the most significant modifiable risk factors for both primary and recurrent hemorrhagic stroke. The American Heart Association recommends maintaining blood pressure below 130/80 mm Hg to reduce the risk of recurrence (7). While several antihypertensive classes are available, agents targeting adrenergic receptors may be more effective than renin–angiotensin system inhibitors for secondary prevention, although further validation is needed (35). Evidence also shows that widespread use of antihypertensive therapy has meaningfully reduced the incidence of hemorrhagic stroke in recent decades.

### Antithrombotic therapy

Antithrombotic management after hemorrhagic stroke remains highly controversial. Anticoagulants and antiplatelet agents can increase bleeding risk, yet withholding them may elevate the risk of thromboembolic events. The REstart or Stop Antithrombotics Randomised Trial (RESTART) suggested that resuming antiplatelet therapy after ICH may not significantly raise the risk of recurrent bleeding compared with avoiding antiplatelets altogether, although its findings are not definitive (35). Current guidelines recommend individualized decision-making based on a patient’s relative risk of ischemic versus hemorrhagic events. Ongoing trials, such as the Antiplatelet Secondary Prevention International Randomized Study After Intracerebral Hemorrhage (ASPIRING), aim to clarify the safety and benefits of antiplatelet therapy in ICH survivors (36). In parallel, widely used antithrombotic agents—including aspirin, warfarin, clopidogrel, and non-vitamin K antagonist oral anticoagulants—continue to play critical roles in preventing recurrent ischemic stroke when carefully selected (37).

### Surgical interventions

Surgical evacuation of hematomas remains one of the primary treatment options for patients with intracerebral hemorrhage, particularly when there is significant mass effect or neurological deterioration. Traditional craniotomy has long been used to remove hematomas; however, its ability to consistently improve long-term neurological outcomes and overall prognosis remains limited (34, 38). As a result, current research has shifted toward identifying patient subgroups that may derive the greatest benefit from targeted surgical intervention, especially those experiencing rapid clinical decline or harboring large, life-threatening hematomas.

Emerging evidence has highlighted the potential of minimally invasive surgery (MIS) as a safer and more effective alternative to open craniotomy. MIS techniques have demonstrated encouraging outcomes, particularly in older patients, where reduced surgical trauma and faster recovery may offer added advantages (39). Methods such as stereotactic aspiration and thrombolysis (SAT) and endoscopic surgery (ES) are increasingly being explored. SAT appears most effective in cases with less severe mass effect, allowing gradual clot dissolution, whereas ES may be more appropriate for larger or more compressive hematomas, where direct visualization and controlled evacuation are essential (40). Current surgical guidelines strongly support intervention for large or compressive cerebellar hemorrhages, but the benefits of surgical evacuation for supratentorial ICH remain uncertain. Ongoing clinical trials continue to investigate whether minimally invasive evacuation strategies can meaningfully improve functional outcomes and expand the therapeutic window for surgical management.

Despite advances in medical and surgical management, current therapies for hemorrhagic stroke remain largely supportive rather than restorative, aimed at limiting hematoma expansion, stabilizing physiology, and preventing complications. However, none of these approaches effectively counteract the progressive secondary injury driven by oxidative stress, neuroinflammation, excitotoxicity, and programmed cell death. As demonstrated in the preceding sections, the cascade initiated by hemoglobin-, heme-, and iron-mediated toxicity unfolds across multiple cellular compartments and persists long after the initial hemorrhage, ultimately impairing neural repair. This therapeutic gap underscores the urgent need for regenerative strategies capable of directly modulating the underlying biology of injury, rather than solely mitigating its consequences.

In this context, cell-based (MSCs) and cell-free biologics (exosomes) have emerged as compelling candidates to complement existing interventions. Given their unique biological properties and growing preclinical evidence, exosome-based interventions may represent a pivotal shift in the treatment paradigm for hemorrhagic stroke—moving from supportive care toward targeted neurorestoration. The following section therefore examines in detail the origins, cargo, biological functions, and therapeutic relevance of exosomes and other extracellular vesicles, laying the groundwork for their potential incorporation into clinical protocols for SAH and ICH.

## Exosomes and other extracellular vesicles

Exosomes are part of the Extracellular vesicles (EVs) group of subcellular membrane-bound particles. EVs are secreted by nearly all cell types, acting as central mediators of intercellular communication in both physiological and pathological contexts. Together with exosomes, EVs are broadly classified into two main categories together with microvesicles (MVs) and apoptotic bodies (APs) based on their size, biogenesis, and cargo. Other less-characterized vesicular structures, such as argosomes and membrane particles, have also been described, further highlighting the complexity of this field (41–43).

Exosomes are the smallest of the EVs, typically ranging from 30/40–100/150 nm in diameter (12, 41–43). They originate as intraluminal vesicles (ILVs) formed within multivesicular bodies (MVBs), which are secreted into the extracellular space when MVBs fuse with the plasma membrane (12, 41–44). Exosomes carry a wide range of bioactive molecules, including proteins, lipids, DNA, mRNA, microRNAs, mitochondrial DNA (mtDNA) and other regulatory RNAs (43, 45–47). Through this diverse cargo, they regulate cell signaling and can reprogram the phenotype and function of recipient cells. Functionally, exosomes have the capacity to participate in immune modulation, tissue regeneration, angiogenesis, and pathogenesis of diseases such as cancer, neurodegenerative disorders, and liver diseases (44, 48, 49). They have been isolated from multiple biological fluids, including blood, urine, cerebrospinal fluid, and breast milk, making them accessible targets for both diagnostic and therapeutic applications (43, 50, 51).

Exosomes arise from the exudation of a variety of cell lines (12, 52). Most cells can naturally produce exosomes including T cells, B cells, natural killer (NK) cells, macrophages, MSCs and others. Nevertheless, exosomes produced by MSCs have special traits that make them a key candidate for therapeutic use (12, 52). These exosomes contain cytokines, signaling lipids, regulatory miRNAs, mRNAs and growth factors that can be beneficial for regeneration purposes (53). A transcriptomic profiling revealed that high-secretor MSCs are enriched for genes involved in EV biogenesis, vascular regeneration, and stem cell proliferation (e.g., RAB27B, TSG101, VEGFA, HGF, CXCL12) (52). Moreover, MSC-derived exosomes are responsive to their microenvironment, and preconditioning strategies can enhance their reparative potential (54). Together, these findings highlight MSCs as an optimal source for therapeutic exosomes, combining intrinsic regenerative cargo with the ability to be bioengineered or primed for disease-specific applications.

MVs are a subtype of EVs ranging from 100 nm to 1 μm in diameter and are formed through the outward budding of the plasma membrane, distinguishing them from exosomes, which originate from the endosomal pathway. These vesicles carry diverse molecular cargo including cytokines, chemokines, lipids, carbohydrates, miRNAs, mitochondria and proteins, allowing them to play active roles in intercellular communication, particularly in the contexts of inflammation, tumor progression, and angiogenesis (47, 55, 56).

Apoptotic bodies are the largest EV subtype, typically 1–5 μm in size, and arise during programmed cell death (apoptosis) (12, 43, 57). They contain fragmented DNA, histones, and other cellular organelles and debris (12, 56, 57). Their primary role is to ensure safe clearance of dying cells while also modulating immune responses through the release of apoptotic signals (12, 57). In addition to exosomes, MVs and APs, other specialized vesicles have been described. Argosomes represent a class of extracellular vesicles implicated in developmental signaling, though they are less well characterized (58). Membrane particles, typically 50–80 nm in size, also bud directly from the plasma membrane and may have specific roles in intercellular signaling (43).

The choice between MVs and exosomes depends largely on the therapeutic context and the biological effect sought. Exosomes, owing to their smaller size and endosomal origin, could be uniquely suited to cross biological barriers such as the BBB, making them especially advantageous for central nervous system applications (12, 59). Indeed, preclinical work has demonstrated that engineered exosomes can deliver therapeutic RNA across the BBB *in vivo*: dendritic cell–derived exosomes modified with the neuron-specific rabies virus glycoprotein (RVG) peptide efficiently transported siRNA to neurons, microglia, and oligodendrocytes, resulting in substantial gene knockdown of targets such as *β*-site amyloid precursor protein-cleaving enzyme 1 (BACE1) in mouse brain (60). These findings underscore the capacity of exosomes, including those derived from MSCs, to possibly cross the BBB but also to serve as potent, non-immunogenic delivery vehicles for therapeutic cargo. By contrast, MVs, generated through direct budding of the plasma membrane, offer a larger size range and a different molecular cargo, broadening their potential in tissue regeneration and paracrine modulation. Both vesicle types are increasingly recognized as powerful tools for drug delivery and cell-free therapies, with ongoing research aimed at optimizing their isolation, standardization, and targeting strategies.

Compared with APs, which primarily serve in the clearance of dying cells and the regulation of immune tolerance, exosomes present therapeutic advantages. They exhibit greater colloidal stability, lower immunogenicity, and the ability to selectively package and deliver bioactive molecules, such as proteins, lipids, and regulatory RNAs, to recipient cells. Importantly, exosomes can be engineered or preconditioned to enhance their regenerative or immunomodulatory effects, features not shared by apoptotic bodies (54). These attributes position exosomes as a more versatile and clinically translatable platform for regenerative medicine, targeted drug delivery, and biomarker discovery.

## Exosome-mediated repair mechanisms in stroke

Exosomes are increasingly investigated as both diagnostic biomarkers and therapeutic agents in stroke. Although most studies remain at the *in vitro* stage, a growing number of *in vivo* models are beginning to elucidate their physiological roles and therapeutic potential. A major advantage of exosomes is their ability to cross—and potentially repair—the BBB, as demonstrated in preclinical studies (15, 60). In their supportive role of angiogenesis, exosomes act as key mediators of intercellular communication, transferring bioactive cargo between endothelial cells, progenitors, and stromal cells, thereby helping to maintain vascular integrity (61). Importantly, exosomes from multiple origins can deliver pro-angiogenic proteins (VEGF, Ang-1, FGF), regulatory lipids, and small RNAs such as miR-21, miR-126, and miR-210, all of which activate pathways including PI3K/Akt, MAPK/ERK, and HIF-1α to stimulate vascular sprouting and repair (19). However, standardized protocols for their clinical application in post-hemorrhagic stroke management have yet to be established. Proposed therapeutic strategies include using exosomes as delivery vehicles for cell-derived factors, engineering them as targeted drug carriers, or applying them directly as transplantation-based therapies (8).

Among these approaches, MSC–derived exosomes have shown particular promise in promoting neurovascular remodeling and neurogenesis, thereby enhancing functional recovery after cerebrovascular injury. In animal models, MSC-derived exosomes stimulate endothelial cell formation, neuronal regeneration, and overall neurological improvement (62–64). Additionally, in rodent models of intracerebral hemorrhage and ischemic stroke, these exosomes attenuate neuroinflammation and support nerve function restoration by modulating key inflammatory pathways, including TLR4/NF-κB signaling (65).

### Angiogenesis

Recent studies underscore the therapeutic relevance of exosome-mediated microRNA delivery in post-stroke angiogenesis and neural repair, with outcomes strongly influenced by the cellular origin of the vesicles and the pathological microenvironment (66). Zhang et al. (66) demonstrated that exosomes derived from bone marrow MSCs (BM-MSCs) engineered to overexpress miR-126 significantly enhanced angiogenesis both *in vitro* and *in vivo*. Using human umbilical vein endothelial cells (HUVECs), the authors showed increased endothelial proliferation, migration, and tube formation, accompanied by upregulation of VEGF and angiopoietin-1, mediated through direct targeting of PIK3R2 and subsequent activation of the PI3K/Akt signaling pathway. *In vivo*, Exo-miR-126 administration increased capillary density and accelerated tissue repair, confirming the angiogenic efficacy of this approach (66).

Complementing these mechanistic findings, Geng et al. (67) reported that exosomes derived from adipose-derived stem cells (ADSCs) enriched in miR-126—an endothelial-associated microRNA found to be downregulated in the plasma of acute ischemic stroke patients—produced robust therapeutic effects in a rat model of transient middle cerebral artery occlusion (tMCAO). Intravenous administration of miR-126–overexpressing ADSC exosomes significantly improved neurological recovery, as assessed by modified neurological severity score (mNSS) and foot-fault testing, while enhancing angiogenesis and neurogenesis, reducing neuronal death, and suppressing microglial activation and pro-inflammatory cytokines, including TNF-*α* and IL-1β, compared with naïve or miR-126–deficient exosomes (67).

In parallel, Chen et al. (68) demonstrated that intravenous delivery of xenogenic adipose-derived MSCs (ADMSCs) or isolated ADMSC-derived exosomes markedly reduced brain infarct volume and preserved neurological function in a rat model of acute ischemic stroke induced by middle cerebral artery occlusion. Through histopathological, magnetic resonance imaging (MRI), molecular, and immunohistochemical analyses, the study revealed attenuation of inflammatory, oxidative stress, apoptotic, and fibrotic pathways, supporting the therapeutic equivalence of cell-free exosome administration to parent stem cell therapy (68).

Supporting the importance of the tissue microenvironment, Lee et al. (69) further showed that microvesicles/exosomes derived from MSCs preconditioned with normal or stroke-injured rat brain extracts exhibited superior efficacy compared with naïve MSC-derived vesicles in a permanent MCAO rat model. Proteomic and immunohistochemical analyses indicated, enhanced angiogenesis, reduced neuroinflammation, and increased endogenous neurogenesis, highlighting that vesicle cargo is dynamically shaped by environmental cues and critically determines therapeutic potency (69).

Collectively, these studies demonstrate that MSC–derived exosomes act as biologically active, microenvironment-responsive carriers of microRNAs and proteins, capable of orchestrating angiogenic, neurogenic, and immunomodulatory responses after stroke. Their efficacy is tightly linked to vesicle origin, cargo composition, and disease context, positioning engineered exosomes as a promising, cell-free therapeutic strategy for cerebrovascular repair.

### Neuroinflammation

Neuroinflammation is a central driver of secondary injury after hemorrhagic stroke, amplifying neuronal death, edema, and long-term functional deficits (70). Emerging evidence suggests that exosomes play a pivotal role in regulating this inflammatory cascade. In a collagenase-induced ICH model, Li et al. (70) demonstrated that inhibition of exosome release using GW4869 markedly aggravated neurological deficits, brain edema, and blood–brain barrier disruption. These effects were associated with enhanced leukocyte infiltration into the brain, whereas transfer of exosomes isolated from ICH brains suppressed inflammatory factor production and reduced injury in recipient mice (70). This highlights that endogenously released exosomes are not bystanders but active modulators of the post-stroke immune response, helping to restrain excessive inflammation and tissue damage.

In 2020, Duan et al. (71) investigated the therapeutic potential of exosomes derived from miR-146a-5p-enriched BM-MSCs in an ICH rat model. Their findings showed that administering exosomes 24 h post-ICH significantly improved neurological function, reduced neuronal apoptosis and degeneration, and suppressed inflammation. These effects were attributed to exosomes downregulating interleukin-1 receptor-associated kinase 1 (IRAK1) and nuclear factor of activated T cells 5 (NFAT5), thereby preventing microglial M1 polarization (71).

More recently, Nan et al. (72) used exosomes derived from human umbilical cord MSC-derived exosomes (hUC-MSC-Exos) to decrease various neuroinflammation related processes and facilitated restoration of nerve function in rats that have intracerebral hemorrhage (72). They concluded that breakdown products of blood cells after ICH as endogenous ligands activate the TLR4/NF-κB inflammatory signaling pathway, leading to the expression of various inflammatory mediators (NF-κB, IL-1β, TNF-*α*) leading to excessive inflammation of the nervous system. hUC-MSCs-Exos blocks this signaling pathway by affecting the expression of TLR4 protein (72).

Taken together, these findings demonstrate that exosomes can reprogram the inflammatory milieu after hemorrhagic stroke, not only dampening harmful immune activation but also creating a permissive environment for subsequent repair. This immunomodulatory capacity paves the way for their role in stimulating regenerative processes, including neurogenesis and angiogenesis, which are critical for long-term recovery.

### Neurogenesis

Neurogenesis—the birth of new neurons from neural stem cells (NSCs)—is a central mechanism of post-stroke brain repair and functional recovery. Following stroke, NSCs from the subventricular zone (SVZ) and the subgranular zone of the dentate gyrus proliferate, migrate toward the infarct and peri-infarct regions, and differentiate into mature neurons that can integrate into existing circuits (73). This endogenous repair process, however, is often insufficient: newborn neurons exhibit limited survival in the hostile microenvironment of the injured brain, where chronic inflammation, oxidative stress, and lack of trophic support impede long-term integration (73). Age further compounds these limitations, with hippocampal neurogenesis in particular being markedly impaired in older animals, highlighting the need for therapeutic strategies that can support and amplify this intrinsic regenerative response.

Exosomes derived from MSCs are increasingly recognized as potent modulators of neurogenesis. Their regenerative effects operate, in part, through antagonizing Prostaglandin E receptor 4 (EP4), a component of the prostaglandin pathway. This action promotes B3-tubulin polymerization and neuronal growth, while the conversion of 2′,3′-cAMP into adenosine provides additional neuroprotective support (74). Importantly, MSC-derived exosomes are enriched with growth factors, miRNAs, and cytokines that directly enhance NSC proliferation, differentiation, and survival within damaged brain regions.

Preclinical models further highlight their therapeutic potential. Exosomes derived from hUC-MSCs engineered to overexpress C-C chemokine receptor type 2 (CCR2) improved post-stroke cognitive impairment by promoting M2 microglia/macrophage polarization, enhancing oligodendrogenesis, and stimulating remyelination (59). Likewise, EVs from NSCs and MSCs administered in thromboembolic stroke models have demonstrated benefits in neurogenesis, functional recovery, and white matter repair (75). Evidence from both ischemic and hemorrhagic stroke models suggests that MSC-derived exosomes may serve as a versatile therapeutic platform capable of addressing diverse injury mechanisms. In ischemic stroke, Xin et al. (76) showed that systemic exosome administration enhanced neurite remodeling, neurogenesis, and angiogenesis, driving coordinated neurovascular repair and functional recovery (76). Similarly, in hemorrhagic stroke, Han et al. (65) demonstrated that exosome treatment improved neurological and cognitive outcomes, while promoting angiogenesis, neurogenesis, and white matter repair in the perihematomal region. These parallel findings indicate that, whether the injury arises from ischemia or intracerebral hemorrhage, MSC-derived exosomes can modulate overlapping regenerative pathways—supporting vascular repair, stimulating neural plasticity, and mitigating secondary tissue loss (65). This dual applicability underscores their potential as a broadly relevant, cell-free therapy across different stroke subtypes.

These findings demonstrate that exosomes not only mitigate neuronal injury but also activate the brain’s intrinsic repair machinery. By enhancing neurogenesis, synaptic plasticity, and neurovascular remodeling, they offer a cell-free therapeutic strategy with the potential to bridge the gap between limited spontaneous repair and the functional demands of stroke recovery. Nonetheless, the timing of administration is likely critical: in clinical settings, delays between the onset of hemorrhage and hospital admission may determine not only survival but also the therapeutic window in which exosome-based interventions can exert maximal benefit.

### Inhibition of apoptosis

Apoptosis is a major contributor to secondary brain injury after hemorrhagic stroke, particularly in the setting of ICH and SAH. Neuronal apoptosis is triggered by several converging pathways, including the extrinsic death receptor pathway, mitochondrial dysfunction, oxidative stress, and endoplasmic reticulum stress, ultimately leading to caspase activation and cell death (77). In SAH, excitotoxicity, blood-derived toxic metabolites, and inflammatory mediators further amplify apoptotic signaling, worsening neurological outcomes. Indeed, irreversible neuronal apoptosis has been recognized as a key determinant of both short- and long-term prognosis after hemorrhagic stroke, highlighting it as a crucial therapeutic target (77).

In 2018 Shen et al. (78) studied the neuroprotective effects of exosomes derived from miR-133b modified MSCs in an ICH rat model. They reported that exosome treatment suppressed RhoA expression while activating the extracellular signal-regulated kinase 1/cAMP response element-binding protein (ERK1/CREB) pathway, which is important for cellular survival and repair. These findings suggest that miR-133b-modified exosomes provide neuroprotection by mediating anti-apoptotic effects through RhoA and ERK1/CREB signaling (78). Similarly, Zhao et al. (79) explored the neuroprotective effects of hUC-MSC-Exos in early brain injury following SAH.

Gao et al. (80) further explored the role of exosomes in SAH using a rat animal model. They found that MSC-derived EVs reduced neuronal apoptosis and improved cognitive function. Their study revealed that SAH-induced upregulation of miR-21 in the prefrontal cortex and hippocampus promoted neuronal survival (80). Additionally, Li et al. (81) reported that miR-137-overexpression enhances the neuroprotective effects of exosomes in oxyhemoglobin-induced apoptosis and mitochondrial dysfunction. This mechanism was mediated by caveolin and clathrin dependent pathways (81). These studies highlight the therapeutic potential of MSC-derived exosomes in promoting recovery and reducing neuronal damage in hemorrhagic stroke models (80, 81). Taken together, these findings indicate that exosomes not only reduce neuronal apoptosis but also reprogram the molecular landscape of injured brain tissue toward survival. This dual action makes them strong candidates for translation into therapies aimed at reducing neuronal loss and promoting recovery in both ICH and SAH.

## MSC-derived exosomes in hemorrhagic stroke: comparison with other cellular sources, advantages, and limitations

Among the different EV sources explored for hemorrhagic stroke, MSCs seem to remain the most extensively studied and translationally advanced parent cell type. Based on the representative comparative literature summarized in Supplementary Table 1, studies on MSC-derived exosomes (72, 82) appear more frequently than those on vesicles derived from neural stem cells (NSCs) (83), ADSCs (84), induced pluripotent stem cells (iPSCs) (85, 86), embryonic stem cells (ESCs) (87), endothelial progenitor cells (EPCs) (81), neural progenitor cells (NPCs) (88), microglia (89), PC12 (transplantable rat tumor with features of neural crest cells) (90) and healthy human plasma collection (91) (PubMed search criteria are provided in Supplementary Table 2). This comparative predominance may reflect the relative ease of MSC isolation and expansion, their established safety profile in regenerative medicine, and the increasing standardization of exosome isolation and characterization methods for MSC-based products.

Across the representative studies summarized in Supplementary Table 1, differential ultracentrifugation was the most commonly employed method for the isolation of MSC-derived exosomes, in some cases combined with multistep centrifugation or ultrafiltration, whereas a smaller number of studies relied on polymer-based precipitation methods such as ExoQuick (65, 75, 78). Likewise, the most frequently used characterization techniques were transmission electron microscopy (TEM), nanoparticle tracking analysis (NTA), and Western blotting for canonical exosomal markers, including CD9, CD63, CD81, and ALIX (65, 76, 78, 79). This degree of methodological convergence is noteworthy, as it suggests that MSC-derived exosomes are supported by a comparatively more reproducible experimental framework than many alternative vesicle sources.

In terms of developmental stage, the representative literature summarized in Supplementary Table 1 remains predominantly preclinical, with most studies combining *in-vitro* and *in-vivo* validation (16, 92, 93). The most commonly used animal platforms were rodent stroke models, particularly middle cerebral artery occlusion (MCAO) for ischemic stroke and ICH or SAH models for hemorrhagic stroke (65, 76, 78). A smaller number of studies extended their evaluation to large-animal models (Yorkshire swine model) of hemorrhagic shock and brain injury, supporting the feasibility of systemic EV administration in more translational settings, although true human clinical studies are still lacking within this group of articles (6, 9, 10, 94–96). Therefore, while MSC-derived exosomes appear to be among the most developed EV platforms in stroke research, their translation remains incomplete.

Another recurrent feature across the literature analyzed in this article is the route of administration. Most studies evaluating MSC-derived exosomes employed intravenous delivery, generally as a single dose administered within hours to 24–72 h after injury (59, 65, 76, 78). For example, BM-MSC-derived exosomes were delivered intravenously at 100 μg 24 h after MCAO in ischemic stroke models (76) and at the same dose 24 h after ICH induction in hemorrhagic stroke models (65). Other studies explored earlier therapeutic windows in SAH, including administration at 5 min, 10 min, or 1 h after injury, whereas some engineered or alternative vesicle platforms used intracardiac, intranasal, intracerebral, or intracerebroventricular routes (7, 11, 12, 15, 20, 74, 79, 82, 92, 97). Together, these observations indicate that dose, timing, and administration schedules remain insufficiently standardized across the current literature.

Despite differences across studies in EV source, experimental design, and preclinical model, several biological outcomes were recurrent. MSC-derived exosomes were frequently associated with enhanced angiogenesis, neurogenesis, white-matter remodeling, and anti-apoptotic signaling, together with reduced neuroinflammation and improved functional recovery (65, 76, 78). In some studies, these effects were linked to specific exosomal cargo, including miR-133b, miR-146a-5p, miR-486-3p and other regenerative miRNAs that may modulate pathways such as RhoA/ERK/CREB, TLR4/NF-κB, and microglial polarization (8, 19, 71, 72, 78, 98, 99). Exosomes from other cellular sources, including NSCs, iPSCs, ESCs, EPCs, NPCs, and microglia, also demonstrated therapeutic potential, particularly as engineered delivery systems or neurotrophic carriers; however, these approaches are currently supported by fewer studies and appear to be less methodologically standardized than MSC-based platforms.

Taken together, the representative evidence included in this analysis suggests that MSC-derived EVs and more specifically exosomes may currently constitute one of the most developed platforms in stroke research, particularly in rodent MCAO, ICH, and SAH models. Their potential advantages include a more established manufacturing background, the recurrent use of commonly accepted isolation and characterization methods, a broader body of *in vivo* efficacy data, and an established parent-cell safety profile in regenerative medicine. However, MSC-derived exosomes also present important limitations, including donor- and tissue source-dependent heterogeneity, variability in cargo composition, incomplete standardization of potency assays, limited biodistribution and persistence data, and the current lack of robust head-to-head comparisons with vesicles from alternative parent cells. Therefore, although MSC-derived exosomes appear especially promising, greater harmonization of dose, treatment schedule, biodistribution assessment, potency testing, and source-specific manufacturing criteria will be necessary before they can be reliably compared with other EV platforms or advanced toward clinical application.

## Conclusion

Hemorrhagic stroke remains one of the most devastating cerebrovascular disorders, with high mortality and profound long-term disability despite advances in acute neurocritical care (1–7). Standard management strategies—centered on blood pressure control, prevention of recurrence, and careful use of antithrombotics—remain insufficient because they do not address the underlying molecular mechanisms of neuronal death, persistent inflammation, and impaired repair (7, 32, 35, 36, 100).

Cell- and subcellular-based therapies, particularly those derived from MSCs, have shown promising neuroprotective and regenerative effects through the modulation of intrinsic repair mechanisms, including the transfer of large cargo such as mitochondria (9, 13, 17, 56, 101, 102). Increasingly, however, it is the cell-free derivatives—exosomes and other extracellular vesicles—that are emerging as the true effectors of paracrine benefit. Exosomes, may have the ability to cross the BBB, stability in circulation, and low immunogenicity, provide a unique therapeutic platform. However, more research is needed regarding better imagin/tracing, molecular dissection (receptor-mediated pathways) and quantitative *in vivo* tracking. Preclinical studies consistently demonstrate that MSC-derived exosomes can reduce neuroinflammation, attenuate oxidative stress, inhibit apoptosis, and stimulate neurogenesis and angiogenesis, ultimately enhancing functional recovery (62, 65, 78, 82, 103–108).

Nevertheless, important translational challenges remain. The therapeutic window is not yet clearly defined. While early administration has shown promise in limiting acute damage, robust evidence is lacking for whether immediate post-hemorrhage delivery translates into superior outcomes (12, 46, 109). Equally, there is little clarity on the impact of repeated dosing, optimal concentration, and long-term administration schedules. These factors are likely to be critical in determining whether exosomes can move from promising preclinical agents to reliable clinical therapeutics. Furthermore, heterogeneity in vesicle cargo, isolation techniques, and manufacturing standards introduces variability that must be resolved before large-scale clinical translation (12, 51, 63, 108, 110).

In this context, MSC-derived exosomes offer more than symptomatic management: they represent a disease-modifying, regenerative approach that could bridge the gap between acute stabilization and long-term recovery in hemorrhagic stroke. Future efforts should prioritize well-designed, multicenter clinical trials that rigorously address dosing strategies, biodistribution, and long-term safety. By systematically overcoming these barriers, exosome-based therapies could reshape the treatment paradigm of hemorrhagic stroke, transforming care from damage containment to true neural repair and functional restoration.

## Data Availability

The original contributions presented in the study are included in the article/Supplementary material, further inquiries can be directed to the corresponding author.

## Supplementary material

Supplementary Table 1 Summary of representative preclinical studies evaluating exosomes and other extracellular vesicle (EV)-based approaches in experimental models of hemorrhagic and ischemic stroke, including study design, parent cell source, isolation and characterization methods, experimental platform, stroke model, route of administration, and dosing schedule. Ref, reference; MSCs, mesenchymal stem/stromal cells; BM-MSCs, bone marrow mesenchymal stem/stromal cells; ADSCs, adipose-derived stem cells; hUC-MSCs, human umbilical cord mesenchymal stem/stromal cells; NSCs, neural stem cells; NPCs, neural progenitor cells; EPCs, endothelial progenitor cells; ESCs, embryonic stem cells; hESCs, human embryonic stem cells; hiPSCs, human induced pluripotent stem cells; hiPSC-MSCs, human induced pluripotent stem cell-derived mesenchymal stem/stromal cells; EVs, extracellular vesicles; sEVs, small extracellular vesicles; iMSC-sEVs, induced pluripotent stem cell-derived mesenchymal stem cell small extracellular vesicles; TEM, transmission electron microscopy; EM, electron microscopy; NTA, nanoparticle tracking analysis; TRPS, tunable resistive pulse sensing; qNano, tunable resistive pulse sensing nanoparticle analyzer; ZetaView, nanoparticle tracking analyzer; Western blot, immunoblot-based protein detection method; ALIX, ALG-2-interacting protein X; TSG101, tumor susceptibility gene 101; GM130, Golgi matrix protein 130; HSP70, heat shock protein 70; HSPA8, heat shock cognate 71 kDa protein; CD9, CD63, CD81, CD90, CD105, CD34, and CD45, cluster of differentiation markers; BDNF, brain-derived neurotrophic factor; CCR2, C-C chemokine receptor type 2; CNP, 2′,3′-cyclic nucleotide 3′-phosphodiesterase; MCAO, middle cerebral artery occlusion; tMCAO, transient middle cerebral artery occlusion; TE-MCAO, thromboembolic middle cerebral artery occlusion; ICH, intracerebral hemorrhage; SAH, subarachnoid hemorrhage; TBI, traumatic brain injury; HS, hemorrhagic shock; OGD, oxygen-glucose deprivation; Nor-N-EVs, normoxic neuron-derived extracellular vesicles; OGD-N-EVs, oxygen-glucose deprivation neuron-derived extracellular vesicles; PBS, phosphate-buffered saline; qRT-PCR, quantitative reverse-transcription polymerase chain reaction; PI3K, phosphoinositide 3-kinase; AKT/Akt, protein kinase B; MCP-1, monocyte chemoattractant protein-1; PTEN, phosphatase and tensin homolog; AMPK, adenosine monophosphate-activated protein kinase; NF-κB, nuclear factor kappa B; STAT3, signal transducer and activator of transcription 3; SIRT1, sirtuin 1; SIRT2, sirtuin 2; NOS2, nitric oxide synthase 2; TXNIP, thioredoxin-interacting protein; TFEB, transcription factor EB; COX2, cyclooxygenase-2; RVG, rabies virus glycoprotein; PC12, rat pheochromocytoma cell line; SH-SY5Y, human neuroblastoma cell line; CA1, cornu ammonis 1.
